# Extracellular Vesicle Associated Non-Coding RNAs in Lung Infections and Injury

**DOI:** 10.3390/cells10050965

**Published:** 2021-04-21

**Authors:** Zhi Hao Kwok, Kareemah Ni, Yang Jin

**Affiliations:** Division of Pulmonary and Critical Care Medicine, Department of Medicine, Boston University Medical Campus 72 E Concord St. R304. Boston, MA 02118, USA; zhihaok@bu.edu (Z.H.K.); kani@bu.edu (K.N.)

**Keywords:** extracellular vesicles, non-coding RNAs, lung infection, lung inflammation, lung injury

## Abstract

Extracellular vesicles (EVs) refer to a heterogenous population of membrane-bound vesicles that are released by cells under physiological and pathological conditions. The detection of EVs in the majority of the bodily fluids, coupled with their diverse cargo comprising of DNA, RNA, lipids, and proteins, have led to the accumulated interests in leveraging these nanoparticles for diagnostic and therapeutic purposes. In particular, emerging studies have identified enhanced levels of a wide range of specific subclasses of non-coding RNAs (ncRNAs) in EVs, thereby suggesting the existence of highly selective and regulated molecular processes governing the sorting of these RNAs into EVs. Recent studies have also illustrated the functional relevance of these enriched ncRNAs in a variety of human diseases. This review summarizes the current state of knowledge on EV-ncRNAs, as well as their functions and significance in lung infection and injury. As a majority of the studies on EV-ncRNAs in lung diseases have focused on EV-microRNAs, we will particularly highlight the relevance of these molecules in the pathophysiology of these conditions, as well as their potential as novel biomarkers therein. We also outline the current challenges in the EV field amidst the tremendous efforts to propel the clinical utility of EVs for human diseases. The lack of published literature on the functional roles of other EV-ncRNA subtypes may in turn provide new avenues for future research to exploit their feasibility as novel diagnostic and therapeutic targets in human diseases.

## 1. Introduction

Respiratory infections, both acute and chronic, are the leading contributors to morbidity and mortality globally [1]. As an essential organ that mediates gas exchange, the lung possesses the second largest surface area of all human tissues that are open to the environment, thereby resulting in direct and constant exposure to inhaled pathogens and noxious stimuli such as allergens and particles. In addition, the intrinsically high levels of oxygen in the lung can have a direct impact on lung immunity and injury. As such, the lung has evolved distinct defense mechanisms for eliciting the appropriate responses to external and internal challenges. As an immune organ system, the lung contains multiple cell types, ranging from immune cells, such as alveolar macrophages (AMs), lung resident dendritic cells (DCs), basophils, and mast cells, to structural cells, such as epithelial cells, which communicate and form an intricate network for regulating immunity.

One of the important sources of cell-to-cell communication is through extracellular vesicles (EVs). Once known as carriers of cellular wastes, emerging evidence has pointed to the indispensable role of EVs in mediating intercellular communication and disease progression via the secretion and release of their cargo molecules [2]. EV cargo represents a diverse load of molecules, including nucleic acids such as DNA, messenger RNA (mRNA) and noncoding RNAs (ncRNAs), as well as proteins that can be transferred from donor to target cells to influence their phenotype. In particular, heterogeneity in the RNA contents of EVs can be observed across different bodily fluids, between vesicles secreted from different cell types, and even among vesicles of the same cell type. As such, ncRNAs have been regarded as one of the most abundant nucleic acids in EVs. Coupled with the detection of EVs in majority of the bodily fluids including saliva, blood, urine, and bile, increasing evidence has pointed to the link between EV-ncRNAs and a variety of physiological as well as pathological processes that may contribute to disease development [3]. In parallel, the diversified ncRNA load of EVs reflecting the intracellular contents of donor cells also makes EVs an attractive tool for diagnostic and therapeutic purposes.

Given the unique features of the lung and the intricate crosstalk between the different cell types in maintaining tissue homeostasis and modulating disease onset, alongside the lack of therapeutic and prognostic targets, identifying and delineating the roles of EV-ncRNAs may potentially shed light on novel therapeutic and diagnostic markers for lung infections and injury. In this review, we summarize the current knowledge on EV-ncRNAs and highlight the functional roles of EV-ncRNAs in lung infections and injury. In addition, we discuss the potential diagnostic and therapeutic values of EV-ncRNAs, as well as some of the challenges faced as we seek to develop them as clinically viable tools. 

## 2. Extracellular Vesicles (EVs)

**Figure 1:**
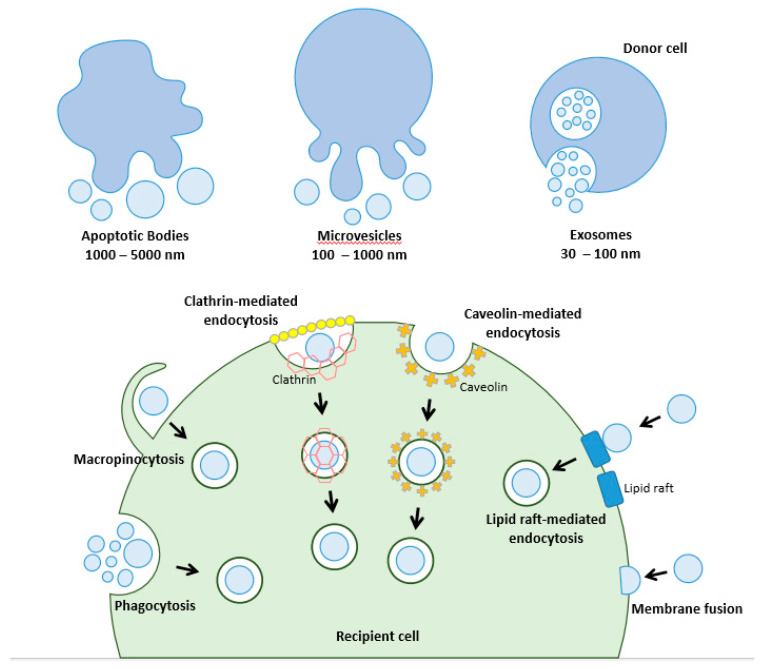
EV subtypes and the routes of EV internalization. EVs can be broadly classified into three categories—namely, the apoptotic bodies, microvesicles and exosomes. Note that these categories are derived from the previous nomenclature [10]. Once secreted from donor cells, EVs can be internalized into recipient cells via multiple pathways, such as clathrin, caveolin, and lipid raft-mediated endocytosis, micropinocytosis, and phagocytosis. Direct membranal fusion between the EVs and plasma membrane of target cells has also been observed.

EVs are a heterogenous population of membrane-bound vesicles containing a wide repertoire of soluble and non-soluble molecules—such as nucleic acids, lipids, and proteins—that are released by cells under physiological and pathological conditions [4]. Based on their physical sizes, biogenesis pathways, and surface markers, EVs have been traditionally classified into three categories: apoptotic bodies (ABs), microvesicles (MVs), and exosomes (Figure 1). ABs are predominantly produced by cells undergoing programmed cell death and are known to be the largest in size amongst the various EV subtypes, with diameters ranging from 1000 to 5000 nm [5]. MVs range from 100 to 1000 nm in size and are typically produced via the direct budding and pinching of the plasma membrane from normal, healthy cells [6]. On the other hand, exosomes are generally formed as multivesicular endosomes (MVEs) upon intraluminal vesicle (ILV) maturation and released via fusion with the cell membrane [7]. Despite these demarcations that have been reported for the differentiation of these EV subtypes, factors such as overlaps in EV surface markers and physical sizes have greatly hampered the use of the above categories to accurately classify EVs. Instead, the International Society of Extracellular Vesicles (ISEV) has set aside guidelines for EV classification as follows: EVs should be defined based on “(a) physical characteristics of EVs, such as size (“small EVs” (sEVs) and “medium/large EVs” (m/lEVs), with ranges defined, for instance, respectively, < 100 nm or < 200 nm [small], or > 200 nm [large and/or medium]) or density (low, middle, high, with each range defined); (b) biochemical composition (CD63+ /CD81+- EVs, Annexin A5-stained EVs, etc.); or (c) descriptions of conditions or cell of origin (podocyte EVs, hypoxic EVs, large oncosomes, apoptotic bodies)” [8,9].

The generation of EVs typically involves the outward blebbing or budding of the plasma membrane. ABs often result from the direct blebbing of the plasma membrane following apoptosis and contain DNA as well as cytoplasmic fragments [5]. For the generation of MVs, budding followed by pinching of the plasma membrane is commonly observed, which can be induced by a variety of intracellular signals, including chemical activation through increased cytosolic Ca^2+^ levels and activation of kinases that regulate actin dynamics, such as the RHO GTPases [11]. Exosomes, on the other hand, were found to originate from endosomal compartments and require a series of intricate mechanisms that mediate the cargo sorting and transportation processes to allow apposition at the cell membrane for budding. The molecular mechanisms governing exosome formation may involve the endosomal sorting complex required for transport (ESCRT) machinery [12], or ESCRT-independent pathways, such as the syndecan/ALIX pathway, ceramide, and tetraspanins [13,14,15].

Once secreted from donor cells, EVs can enter bodily fluids and be taken up by distant recipient cells, leading to changes in molecular processes or signaling that may alter their phenotypes. The interaction of EVs with target recipient cells occurs mainly through specific binding to surface receptors and the subsequent internalization and release of the cargo load. The recognition and initial docking of EVs onto the surface of target cells involve protein–protein interactions between EV surface markers and membrane receptors or contact proteins of recipient cells. Proteins that were found to mediate such interactions include proteoglycans, lectins, and integrins [16]. Critically, recent evidence has pointed to the role of post-translational modifications (PTMs) of these interactors in influencing the eventual uptake of EVs [17]. Once bound to the surface, EVs can then be internalized into the cells, predominantly via an endocytic pathway. The precise mechanisms governing this process, however, remain highly debatable. A variety of molecular mechanisms, including clathrin-mediated endocytosis (CME), caveolin-dependent endocytosis (CDE), phagocytosis, micropinocytosis, and direct membranal fusion, have been reported to regulate EV uptake (Figure 1) [16]. The involvement of lipid raft proteins in EV internalization has also been illustrated [18]. Similarly, the specificity of EV uptake is yet to be fully understood. Emerging evidence suggests that EV uptake is a highly specific process, suggesting the existence of tightly controlled regulatory mechanisms that may involve the “right” configuration of protein–protein interactions to confer selective EV internalization [19,20,21,22,23]. EV uptake can also be influenced by the nature and characteristics of these nanoparticles [24], as well as dynamic factors such as the metabolic state of recipient cells [23]. Nevertheless, these studies shed light on this complex process and how cells may govern preferential uptake of EVs. Critically, these findings point to the inherent repertoire of regulatory pathways that cells possess, which favor uptake, and to the varying combinations of these mechanisms, which can be adopted by different EVs and target cells.

### EV Cargo

The diverse contents across EVs may often reflect the differential loading of the different types of cargo based on cell types, physiological, and pathological conditions, as well as mode of EV biogenesis. Owing to advances in sequencing technologies, we now have an increasing number of publicly available databases with deposited data on the protein, nucleic acids, and lipid contents of EVs, alongside the respective isolation and purification methods performed. These valuable resources will inevitably aid in the unravelling of the molecular mechanisms underlying the heterogeneity of EV cargos observed. 

Some of the commonly enriched proteins in EVs include components of the ESCRTs and those that are involved in the various pathways responsible for EV biogenesis, such as the RAB family of proteins, tetraspanins, as well as certain transmembranal proteins [25]. The lipid composition of EVs was similarly found to exhibit common features with that of the donor cells, with lipids such as sphingomyelin, ceramide, cholesterol, and phosphatidylserine often found enriched as a result of their participation in EV biogenesis [25]. 

As opposed to proteins and lipids, the nucleic acid contents of EVs have been reportedly found to exhibit a different profile than that of the donor cells [26,27]. Specific RNAs, for instance, were shown to be only enriched in the EVs, thus hinting at the existence of highly selective loading mechanisms for nucleic acids (at least for RNAs). Nevertheless, a broad range of nucleic acids, including genomic and mitochondria DNA fragments, small RNAs such as microRNAs (miRNAs), rRNAs (ribosomal RNAs), tRNA fragments, piwi-interacting RNAs (piRNAs), messenger RNAs (mRNAs), and long noncoding RNAs (lncRNAs), have been identified in EVs [28,29,30].

## 3. Non-Coding RNAs (ncRNAs)

**Figure 2:**
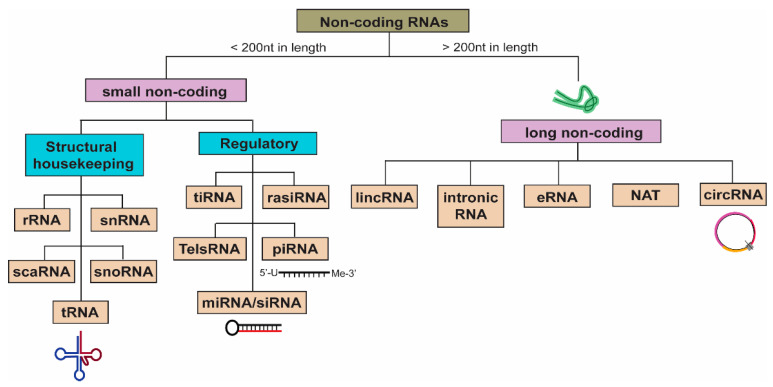
Classification of non-coding RNAs. Non-coding RNAs can be generally divided into two groups, the small non-coding (<200 nucleotides in length) and long non-coding RNAs (>200 nucleotides in length). The former category comprises structural RNAs such as ribosomal RNA (rRNA), small nuclear RNA (snRNA), and transfer RNA (tRNA), while long non-coding RNAs include long intergenic (lincRNA) and intronic RNA, enhancer RNA (eRNA), natural antisense transcripts (NAT), and circular RNA (circRNA). To date, the predominant subtypes of non-coding RNAs that have been commonly found in EVs include rRNA, miRNAs, as well as lincRNAs, intronic RNAs, and circRNAs.

Non-coding RNAs (ncRNAs) represent a diverse class of RNAs with no to little coding potential. Once considered as “junk” DNA, these transcripts have since been extensively studied for their key regulatory functions, which mediate important cellular processes to affect the physiological and pathological states of cells. ncRNAs can be broadly classified into two categories: small ncRNAs that are less than 200 nucleotides in length and long ncRNAs (lncRNAs) with nucleotide length exceeding 200 bases. Small ncRNAs include structural RNAs such as rRNA and tRNA, as well as regulatory RNAs such as miRNAs and piRNAs (Figure 2). While rRNA and tRNAs are essential for housekeeping processes such as ribosomal formation and de novo translation, miRNAs and, more recently, piRNAs have been widely implicated in the regulation of gene expression [31,32]. 

**Figure 3:**
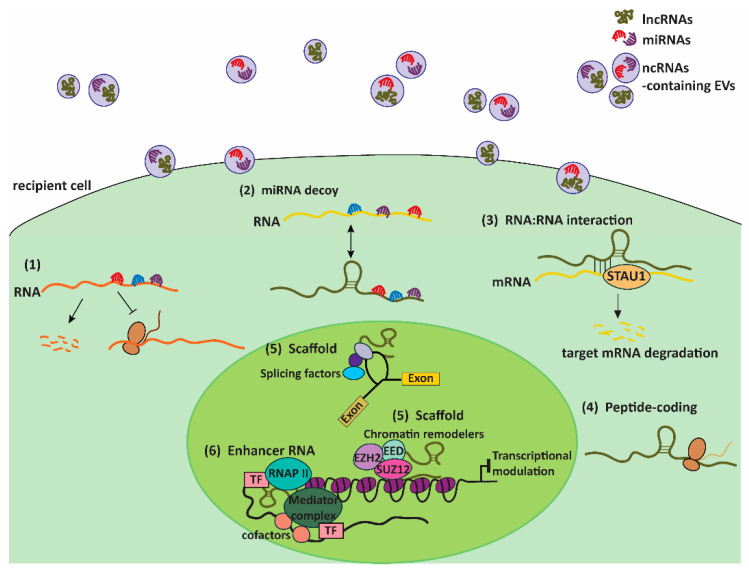
Functional modalities of ncRNAs (specifically microRNAs and long non-coding RNAs) in cells. Upon internalizing into recipient cells, microRNAs (miRNAs) and long non-coding RNAs (lncRNAs) can regulate a variety of molecular processes to induce changes in cell phenotype. (**1**) MiRNAs can bind to complementary sequences on target RNA transcripts via their seed sequences, leading to either transcript degradation (perfect sequence complementarity) or translation inhibition (imperfect sequence complementarity). (**2**) LncRNAs can function as miRNA decoys by sequestering the miRNAs that are common to other RNA transcripts. By doing so, they can positively regulate the expression of these transcripts. (**3**) Through RNA:RNA interactions, lncRNAs can modulate the expression of messenger RNAs (mRNAs) by mediating the Staufen1 (STAU1)-mediate decay (SMD) of target mRNAs. (**4**) LncRNAs may possess multiple open reading frames and thus encode for small, functional peptides. (**5**) LncRNAs can act as scaffolds by forming docking sites for protein complexes involved in molecular processes, such as splicing and chromatin remodeling. (**6**) Enhancer RNAs can activate transcription of target genes by acting as guided loops bound by transcription factors (TFs), cofactors, and RNA polymerase II (RNAP II) to engage distal promoters.

MiRNAs, approximately 20–25 nucleotides in length, are predominantly intronic, i.e., processed from the intronic regions of their host transcription units and thus share similar expression patterns and regulatory mechanisms with their host genes [33]. Based on sequence complementarity, the recognition and binding of miRNA seed sequences to target transcripts modulates the expression of target genes in a RISC-dependent manner via transcript degradation (perfect sequence complementarity) or by inhibiting translation (imperfect sequence complementarity) (Figure 3) [31]. As such, miRNAs consequently exert functional effects in a wide range of cellular processes, such as proliferation and apoptosis, as well as the modulation of immunologic responses that are frequently dysregulated in respiratory infections and injury. For instance, miR-23a and miR-155 were found to be upregulated upon bacterial infections, with implications in bacterial survival in infected lung macrophages and interleukin-17 and -23 production for bacterial clearance, respectively [34,35]. In acute lung injury (ALI) and chronic obstructive pulmonary disease (COPD), miR-34b and miR-195 have been shown to regulate inflammation and proliferation by targeting progranulin (PGRN) to modulate proinflammatory cytokine production and PH domain leucine-rich repeat protein phosphatase-2 (PHLPP2), respectively, to affect Akt signaling [36,37]. 

On the other hand, lncRNAs have a more diverse range of functional modalities, and they include intergenic RNAs or intronic RNAs, enhancer RNAs (eRNAs), natural antisense transcripts (NATs), and circular RNAs (circRNAs) (Figure 2). Over the past decade, extensive research has since unraveled increasing mechanistic roles of lncRNAs, such as miRNA decoy, scaffold for protein complexes to affect transcription and RNA splicing, molecular guide for chromatin remodeling, RNA:RNA interactions to modulate staufen1-mediated decay (SMD), as well as translation of small functional peptides (Figure 3) [38]. One of the first and most well-studied lncRNA is *Xist*, a 17-kb lncRNA essential for the initiation of X chromosome inactivation (XCI) by recruiting chromatin remodeling complexes, such as the polycomb repressive complexes (PRC1 and PRC2), to facilitate histone methylation (H3K27me3) for gene silencing [39]. Recent studies have also demonstrated that lncRNAs containing multiple open reading frames are capable of producing small peptides [40]. One such functional peptide encoded by a lncRNA is the 90-amino acid-long SPAR, found to be conserved in human and mouse, which was shown to negatively regulate mTORC1 activation and suppress muscle regeneration [41]. In the context of lung injury such as ALI, lncRNA FOXD3-AS1 was found to promote lung epithelial cell death and growth inhibition by sequestering miR-150 [42]. LncRNA H19 was demonstrated to promote fibrosis progression by stimulating fibroblast proliferation and increased collagen deposition via the miR-196a/COL1A1 axis, contributing to the onset of idiopathic pulmonary fibrosis (IDF) [43].

## 4. EVs: Couriers of ncRNAs

The RNA cargo of EVs was believed to reflect at least in part the transcriptomes of donor cells. However, increasing data generated from sequencing of EV content indicated that the ncRNA profiles of EVs differ significantly from those of the donor cells, providing a basis for the preferential incorporation of selective ncRNA species into EVs. As illustrated by a recent RNA-sequencing of small RNAs in EVs derived from five cell lines (namely, HEK293T, RD4, C2C12, Neuro2a, and C17.2), rRNA fragments represented 30–94% of the total reads, while miRNAs accounted for 15–80% of the remaining read counts [44]. On top of the differences in RNA species, studies have also shown that these selective RNA molecules are loaded into EVs at different efficiencies. For instance, earlier studies demonstrated that specific miRNAs are preferentially packaged into EVs [45,46], while a recent study showed that tRNA fragments are favorably loaded into EVs as opposed to miRNAs during T cell activation [47]. The increase in generation of these high-throughput data, together with the enhanced discrepancies in EV-RNA profiles owing to the different physiological and experimental conditions of donor cells investigated, warrants the development of computational approaches to systematically sort through this complex load of information. To this end, several databases, such as the exoRBase (http://www.exoRBase.org), which contains mainly the non-coding RNA data generated from sequencing analysis of human blood exosomes [48], as well as the EVmiRNA (http://bioinfo.life.hust.edu.cn/EVmiRNA#!/), which contains deposited miRNA sequencing profiles in EVs [49], have been developed over the recent years. 

Several mechanisms involving sequence specificity, chemical modifications, secondary structures, affinities for membranal lipids, and interactions with RNA binding proteins (RBPs) have since been proposed to account for the highly controlled and specific process of selective ncRNA packaging into EVs. Certain miRNAs, for example, were found to be selectively sorted into EVs through the interaction with the RBP hnRNPA2B1 via the EXOmotif (GGAG), which may be sumoylated to further promote its binding to these miRNAs and the subsequent localization into exosomes [50]. Alternatively, the O-GlcNAcylation of hnRNPA2B1 by caveolin-1, a membrane-bound protein, was shown to enhance its binding to miR-17/93 and direct them into MVs [51]. In addition to RBPs, the 3′ end post-transcriptional modifications of miRNAs can also influence their selective loading into EVs. For example, miR-2909 was found to be targeted to or away from exosomes depending on its 3′ end adenylation to uridylation ratio, linking it to the differential distribution of adenosine kinases across donor cells and exosomes [52]. Selective miRNA export via EVs can also be attributed to the activity of lipid-associated pathways. For example, the inhibition of sphingosine kinase 2, and consequently sphyngosine-1-phosphate synthesis, was shown to impede loading of miR-21 into exosomes [53]. 

Apart from miRNAs, lncRNAs have also been found to be selectively enriched in EVs. One such example is the significantly elevated levels of lincRNA-p21, a lowly abundant intracellular lncRNA, in small EVs derived from cell lines that undergo genotoxic stress [54]. CircRNAs, a subset of lncRNAs, were similarly found to be not only significantly enriched in EVs as compared to donor cells but also more abundant than their linear counterparts in those vesicles [55,56]. While little is known about the mechanisms governing the specific incorporation of lncRNAs into EVs, they are believed to involve the same overlapping molecular machineries as those that regulate mRNA loading. With the identification of consensus motifs such as ACCAGCCU, CAGUGAGC, and UAAUCCCA on the 3′ untranslated regions of mRNAs that allow binding to RBPs such as YBX1 for sorting into EVs, lncRNAs containing these sequences may similarly be directed into EVs [57,58]. The existence of these selective packaging mechanisms for ncRNAs may thus allow for some of the concerted adaptative cellular responses to external stimuli, such as the induction of inflammatory responses to bacterial infection [59] and the facilitation of intercellular crosstalk by protecting these important molecules from extracellular degradation.

Upon internalization into recipient cells and subsequent escape from degradative pathways, EV-ncRNAs may then act to modulate important cellular processes to elicit a functional response. To date, a majority of the studies on EV-ncRNAs have focused on EV-miRNAs, with limited data on the functions of other EV-ncRNA species in target cells. EV-mediated functional transfer of miRNAs has been widely reported for a variety of physiological changes in cell states and diseases. One of the more prominent examples is the astrocytes-derived EV-miR-19, which suppressed the levels of the key tumor suppressor PTEN in recipient cancer cells, leading to the increase in brain metastases [60]. More recent examples of EV-miRNAs with angiogenic and immunomodulatory effects include glioma-derived EV-miR-9 in inducing reprograming of recipient endothelial cells to promote angiogenesis [61], as well as miR-21 in stimulating the proliferation of microglia, a group of innate immune cells in the central nervous system, by downregulating the expression of Btg2 [62]. 

In the case of EV-lncRNAs, recent studies have demonstrated the functional role of EV-mediated transfer of lncRNAs in various biological processes. For example, hypoxic cardiomyocytes-derived EV-lncRNA *NEAT1* was shown to induce a reprogramming of recipient fibroblasts favoring the profibrotic phenotype [63]. In a separate study, tumor-associated macrophages-derived EV-lncRNA *HISLA* was demonstrated to induce aerobic glycolysis and apoptotic resistance of recipient breast cancer cells, leading to further aggravation of the disease [64]. 

With the improvements in RNA-sequencing depth and microarray platforms for EVs, findings to support the functional roles of various EV-ncRNA species will certainly be evident in the near future. Apart from elucidating the functional transfer of different EV-ncRNAs subtypes, a related research topic in the EV field awaiting further exploration is the role of intracellular ncRNAs in regulating EV biogenesis, secretion, and uptake across different physiological and pathological conditions. Advances in this topic will no doubt provide an extra avenue for the development of novel therapeutic targets for the respective diseases.

## 5. EV-ncRNAs in Lung Infections and Injury

To date, a majority of the research on EV-ncRNAs in lung infections and injury focused on EV-miRNAs, with little to none on other EV-ncRNA species. Hence, we summarize in this section of the paper the current state of knowledge on EV-miRNAs in the various types of lung infection and injury (Table 1).

### 5.1. Bacterial Infections

Pneumonia is a respiratory tract infection caused by inhalation of bacteria, fungi, or viruses. Patients with chronic lung diseases, such as COPD and cystic fibrosis, and respiratory infections, such as influenza, or chronic illnesses, such as heart disease, are more prone to pneumonia. Common bacterial pathogens of pneumonia include *Streptococcus pneumoniae, Pseudomonas aeruginosa,* and *Klebsiella pneumonia* [78].

In the case of *Pseudomonas aeruginosa* infection in the lung, bronchoalveolar lavage fluids (BALF)-derived EV-miRNAs were reported to promote innate immune responses such as M1 macrophage polarization, neutrophil recruitment, and pro-inflammatory cytokine production in recipient alveolar macrophages [79]. Future studies could examine the precise role of specific EV-miRNAs in *P. aeruginosa*-induced lung inflammation. In response to *Klebsiella pneumonia* infection, miRNA-223/142 levels were dramatically increased in MVs isolated from BALF and serum [59]. Mechanistically, these MV-miRNAs suppress the activation of the Nlrp3 inflammasome in macrophages, leading to the inhibition of proinflammatory responses induced by the infection [59]. Taken together, these studies clearly demonstrated the functional roles of EV-miRNAs in mediating downstream inflammatory responses in recipient cells and highlighted the potential of these molecules as novel diagnostic markers for pneumonia.

### 5.2. Viral Infections

Common viral causes for pneumonia include influenza viruses, adenoviruses, respiratory syncytial virus (RSV), parainfluenza viruses, and coronaviruses [80]. Infection of human lung adenocarcinoma epithelial A549 cells with influenza virus was reported to induce an elevated amount of intracellular miR-1975 and the subsequent packaging of the miRNA into exosomes [65]. Upon its internalization by recipient cells, exosome-miR-1975 suppressed influenza virus replication, suggesting that it may contribute to the host defense against viral infection [65]. In another study, BALF exosomes containing miR-483-3p, miR-374c-5p, and miR-466i-5p upregulated the expression of pro-inflammatory cytokines (TNF-alpha, interleukin-6, C-C motif chemokine ligand 2), SP100, and interferon-stimulated genes in recipient cells upon influenza viral infection [66]. Mechanistically, miR-483-3p was found to target RNF5 and CD81, which in turn inhibited RIG-1 signaling and resulted in the increased expression of type I interferon and pro-inflammatory cytokines [66]. Overall, these findings highlight the functional transfer of exosomal miRNAs in eliciting immune responses to influenza infection.

In contrast to other types of viral pneumonia, adenovirus pneumonia is an upper respiratory tract infection characterized by a 50% fatality rate [81]. Analysis of exosomal miRNAs from adenovirus pneumonia-derived serum showed that the expression levels of miR‑450a‑5p/miR‑103a‑3p and miR‑103b‑5p/miR‑98‑5p are elevated as compared with those in exosomes derived from normal control serum [67], suggesting the diagnostic value of these miRNAs for the disease.

Coronavirus disease 2019 (COVID-19) is caused by infection with severe acute respiratory syndrome coronavirus 2 (SARS-CoV-2). Recent studies suggest that exosomes may play a role in internalization of SARS-CoV2 virus [82]. Infection of the virus was shown to increase the production of exosomes that may transfer SARS-CoV-2 receptor to recipient cells and consequently promote the binding of SARS-CoV-2 to these cells [82]. While little is known about the role of EV-miRNAs in mediating SARS-CoV infection, host miRNAs were found to exert crucial roles in regulating viral replication and pathogenesis [83]. Upon infection with SARS-CoV, mice lungs displayed elevated levels of miR-21-3p, which was subsequently demonstrated to exhibit the highest probability of binding to the viral RNAs [83]. Further examination of miR-21-3p levels in secreted EVs may elucidate the potential functions of this miRNA in SARS-CoV infection.

Tuberculosis (TB) is a disease caused by bacteria, *Mycobacterium tuberculosis*, which spread through air and infect the lung. *Mycobacterium tuberculosis* infection in macrophages could suppress immune surveillance and the incorporation of inflammation-associated miRNAs into exosomes [84]. Human monocyte-derived macrophages infected with *Mycobacterium bovis* bacillus Calmette–Guérin (BCG) were shown to release exosomal miRNAs such as miRs-1224, -1293, -425, -4467, -4732, -484, -5094, -6848, -6849, -96, and –4488, most of which are generally involved in metabolism and cell signaling pathways [68]. Further analysis of serum from TB patients reveals significantly elevated levels of exosomal miR-484, -425, and -96, which are involved in metabolism, immune system regulation, and signaling pathways [69].

### 5.3. Acute Lung Injury (ALI) and Acute Respiratory Distress Syndrome (ARDS)

Acute respiratory distress syndrome (ARDS) is a potentially lethal acute inflammatory lung condition that affects up to three million people annually and contributes to 24% of cases whereby patients in intensive care units are being ventilated [85]. Currently, there are no known treatments that specifically target its pathophysiology. Mesenchymal stem cell (MSCs)-based therapy has emerged as a promising approach owing to its ability to target the pathophysiological aspects of ARDS. However, issues with relying on MSCs whole cell administration—including their potential tumor-promoting effects, immunogenicity, as well as reduced efficacies due to repeated freeze-thaw cycles—have generally impeded its further development as a viable therapeutic option [86,87]. As MSCs are well-established as conferring protective paracrine effects, which are in part mediated by EV secretion, increasing research has thus actively explored the harnessing of MSC-derived EVs as a cell-free therapeutic option. Since then, several studies have identified the relevance of MSC-derived EV-miRNAs in modulating key pathophysiological processes underlying ARDS in both in vitro and in vivo models of ALI. For example, a recent study revealed the cytoprotective effects of MSC-derived exosomal miR-21-5p against oxidative stress-induced cell death in murine lung ischemia/reperfusion (I/R) and in vitro hypoxia/reoxygenation (H/R) models [70]. Mechanistically, miR-21-5p was found to target phosphatase and tensin homolog (PTEN) and programmed cell death 4 (PDCD4), leading to the amelioration of both intrinsic and extrinsic apoptotic pathways [70]. In a separate study, MSC-derived exosomal miR-30b-3p was shown to similarly promote the proliferation of recipient type II alveolar epithelial cells by targeting serum amyloid A-3 (SAA3) to inhibit apoptosis [71]. Taken together, these data strengthened the viability of developing MSC-EVs and their miRNA cargo as novel therapeutic strategies for ARDS.

### 5.4. Chronic Lung Injury

Chronic obstructive pulmonary disease (COPD) refers to a group of progressive chronic diseases that cause airflow blockage and breathing difficulty. The most common COPD conditions are emphysema, which is damaged alveoli, and chronic bronchitis. Current treatments are limited to bronchodilators and inhaled corticosteroids. Identifying novel EV-ncRNAs that play a functional role in this disease model may pave the way for the development of novel therapies and treatments. Recent studies show that EV-mediated intracellular crosstalk contributes to COPD pathogenesis. For example, expression of miR-210 was increased intracellularly as well as in the EVs derived from COPD samples [72]. Specifically, miR-210 from human bronchial epithelial cells (HBECs)-derived EV was found to promote the differentiation of lung myofibroblasts by targeting autophagy-related 7 (ATG7), leading to reduced autophagy and increased myofibroblast accumulation in COPD pathogenesis [72]. Similarly, miR-21 derived from the exosomes of HBECs were also shown to be elevated in expression in COPD patients and to contribute to myofibroblast differentiation via the epithelium-fibroblast crosstalk [73]. Collectively, these studies indicated that EV-miR-21 and miR-210 may potentially be developed as valuable biomarkers and therapeutic targets for COPD.

Asthma refers to a group of chronic inflammatory diseases resulting from nonspecific stimuli in the lung airways. The major contributors to the pathogenesis of asthma are namely airway inflammation and remodeling, as well as hyperresponsiveness [88]. Despite the increasing findings that indicate the relevance of EV-miRNAs expression in the development of asthma and as potential diagnostic markers, most studies did not further examine the underlying mechanistic roles of these miRNAs in asthma-related cellular processes. For instance, studies measuring exosomal miRNA profiles in asthmatic patients and healthy controls reported the specific alteration in expression levels of miRNAs such as let-7a, miRNA- 21, miRNA-658, miRNA-24, miRNA-26a, miRNA-99a, miRNA-200c, miRNA-1268, miR-1827, miR-346, and miR-574-5p [74,75]. In another study, differential expression of 23 miRNAs was observed in the serum exosomes isolated from rats with airway inflammation induced by zinc oxide particles as compared with that from healthy rats [89]. Further in silico analyses revealed that these differentially expressed miRNAs may be involved in pulmonary inflammation processes [89].

In patients with idiopathic pulmonary fibrosis (IPF), a chronic lung disease with unknown causes, serum EVs were found to contain increased levels of miR-21-5p [76]. Further analysis of this miRNA after a 30-month follow-up period in the patients revealed that miR-21-5p was significantly associated with increased death risk and mortality [76]. In contrast, patients treated with pirfenidone, a medication commonly administered as IPF treatment, were found to exhibit a reduced expression of serum EV miR-21-5p or consistently lower levels of the miRNA in their serum EVs [76], strongly suggesting the relevance of miR-21-5p as a prognostic marker for predicting treatment responsiveness for IPF.

Bronchopulmonary dysplasia (BPD) is a chronic lung disease commonly linked to newborn prematurity [90]. Symptoms of BPD include damaged bronchi and tissue destruction in the alveoli. Exosomes from tracheal aspirates of infants with severe BPD, as well as BALF of hyperoxia-treated newborn mice, were shown to have reduced expression of miR-876-3p [77]. Mechanistically, the reduction was accompanied by an elevated level of miR-876-3p targets, resulting in an aggravated alveolar hypoplasia [77], suggesting a causal relationship between miR-876-3p and BPD pathogenesis.

### 5.5. EV-miRNAs as Diagnostic and Therapeutic Tools in Lung Infections and Injury: Promises and Challenges

With compelling evidence pointing to the differential expression and functional relevance of specific EV-miRNAs in the pathophysiology of lung infections and injury, their potential as novel diagnostic and therapeutic approaches has garnered immense interest within the regenerative medicine field for these diseases. Moreover, the concerns of employing MSCs-based therapies for a variety of lung diseases (mentioned in the previous section) have further propelled the development of MSCs-derived EVs as alternative therapeutic molecules. To date, there has been a growing number of studies that have demonstrated the efficacy of administrating MSCs-derived EVs for a variety of lung infections and injuries [91]. Notably, some of the MSCs-derived EV-miRNAs were shown to exhibit significant biological effects for the alleviation of these respiratory diseases [92]. For example, MSCs-derived exosomal transfer of miR-27a-3p was shown to effectively reduce the levels of proinflammatory cytokine TNF-alpha and induce the polarization of macrophages toward the M2 phenotype, leading to the alleviation of acute lung injury [93]. In a separate study, mesenchymal stem cell (MSC)-derived EV-miR145 was found to reduce the severity of bacterial *E. coli* pneumonia by suppressing multidrug resistance-associated protein 1 (MRP1) protein levels and increasing phagocytosis of *E. coli* by the recipient macrophage cells [94]. While overexpressing miR-145 resulted in increased phagocytosis of bacteria by the target cells, the downregulation of miR-145 in MSC-EVs resulted in reduced anti-inflammatory effects [94].

Despite these encouraging results, much more remains to be investigated and resolved before EV-ncRNA-based therapies can be fully realized for clinical purposes. Firstly, the robust isolation and characterization of different EV subclasses from the lung fluids can be challenging due to technical limitations and lack of consensus in the separation and purification methods for these nanoscale vesicles. Repeated rounds of isolation to attain consistency in their characterization may therefore require repeated invasive harvesting of these fluids. In the context of EV RNA cargo, a systematic approach to obtain a comprehensive catalogue of these cargos—which requires significant improvements in the state-of-the-art sequencing and microarray platforms—will be necessary because diverse RNA subclasses are increasingly being identified in the EVs. After all, these various ncRNA species are mostly low in abundance, and expression levels can vary greatly across different EV subtypes and even “similar” EVs from the same donor cell types. Assuming that these issues can be addressed, factors such as scalability of the methods, manufacturing, and regulations will then need to be established and optimized for translational use.

Additionally, the rate of EV uptake, as well as degradation rate of these EV-ncRNAs (assuming that they are “efficiently” internalized into recipient cells) in part due to the various endosomal escape routes, are the limiting factors in ensuring the functional delivery of these cargos. Moreover, the manipulation of expression levels of specific ncRNAs to be loaded onto the EVs is experimentally challenging to optimize and control, and undesirable levels of these molecules may lead to either the inadequate therapeutic efficacy or unwanted side effects in vivo. Finally, the doses and routes of administration for each lung disease are yet to be determined. The doses and delivery routes varied across the different studies that were described, and critically, the pharmacokinetics of EV-miRNAs remain to be thoroughly investigated. For example, significant therapeutic effects were observed with a single dose of EVs in several studies, while others reported multiple repeats of doses to achieve consistent and superior effects.

## 6. Future Perspectives

With clear evidence pointing toward the significant roles of EV-ncRNAs in affecting cellular processes that may contribute to lung diseases, much more remains to be learned about the regulatory mechanisms governing the selective incorporation of specific ncRNA species and their degradation as EV cargo in recipient cells. Improvements in both computational and experimental approaches are necessary to shed light on the complex biology of EV-ncRNAs. With consistent advances in research technologies, knowledge of these molecular processes will no doubt be increasingly uncovered. In parallel, the booming field of employing nanoparticles as vectors for the specific and efficient delivery of RNAs to target cells has significant implications for the engineering of EVs for therapies. By leveraging the properties of both these tools, the systematic and robust delivery of nucleic acids as therapeutics will inevitably be attainable in the near future.

## Figures and Tables

**Table 1:** Examples of EV-miRNAs with reported functions and significance in lung infection and injury.

Lung Diseases	EV-ncRNA	Possible Function(s)/Significance	References
Bacterial infection	BALF-MV miRNA-223/142	Potential biomarker for lung inflammation and macrophage activation.	[59]
Influenza viral infection	Exosomal hsa-miR-1975	Suppresses influenza virus replication in recipient cell.	[65]
	BALF-exosomal miR-483-3p	Promotes innate immune response against influenza.	[66]
Adenovirus infection	Serum–exosomal miR‑450a‑5p/miR‑103a‑3p and miR‑103b‑5p/miR‑98‑5p	Potential biomarker for adenovirus pneumonia.	[67]
TB	Human monocyte-derived macrophages-exosomal miR-1224, -1293, -425, -4467, -4732, -484, -5094, -6848, -6849, -96, and -4488	Plays a role in host–pathogen interaction.	[68]
	Serum-exosomal miR-484, -425, and -96	Potential biomarkers for TB diagnosis.	[69]
ALI/ARDS	MSC-exosomal miR-21-5p	Targets PTEN and PDCD4 to inhibit apoptosis.	[70]
	MSC-exosomal miR-30b-3p	Targets SAA3 to inhibit apoptosis.	[71]
COPD	HBEC-derived EV miR-210	Promotes differentiation of lung myofibroblasts.	[72]
	HBEC-derived exosomal and serum exosomal miR-21	Potential biomarker for COPD.	[73]
Asthma	BALF-exosomal let-7a, miRNA- 21, miRNA-658, miRNA-24, miRNA-26a, miRNA-99a, miRNA-200c, miRNA-1268, miR-1827, miR-346, and miR-574-5p	Potential biomarkers for asthma.	[74,75]
IDF	Serum-EV miR-21-5p	Potential biomarker for treatment responsiveness.	[76]
BPD	BALF-exosomal miR-876-3p	Potential biomarker for BPD.	[77]

ALI = acute lung injury; ARDS = acute respiratory distress syndrome; BALF = bronchoalveolar lavage fluid; BPD = bronchopulmonary dysplasia; COPD = chronic obstructive pulmonary disease; HBEC = human bronchial epithelial cells; IDF = idiopathic pulmonary fibrosis; MPR1 = multidrug resistance-associated protein 1; MSC = mesenchymal stem cells; PDCD4 = programmed cell death 4; PTEN = phosphatase and tensin homolog; SAA3 = serum amyloid A-3; TB = tuberculosis.

## Data Availability

Not applicable.
